# Human Immunodeficiency Virus Proteins Mimic Human T Cell Receptors Inducing Cross-Reactive Antibodies

**DOI:** 10.3390/ijms18102091

**Published:** 2017-10-03

**Authors:** Robert Root-Bernstein

**Affiliations:** Department of Physiology, Michigan State University, 567 Wilson Road, Room 2201, East Lansing, MI 48824, USA; rootbern@msu.edu; Tel.: +1-517-884-5039

**Keywords:** acquired immunodeficiency syndrome, antigenic mimicry, lymphocytotoxic autoantibodies, autoimmunity, holoimmunity, autoholoimmunity, microbiome, tolerance

## Abstract

Human immunodeficiency virus (HIV) hides from the immune system in part by mimicking host antigens, including human leukocyte antigens. It is demonstrated here that HIV also mimics the V-β-D-J-β of approximately seventy percent of about 600 randomly selected human T cell receptors (TCR). This degree of mimicry is greater than any other human pathogen, commensal or symbiotic organism studied. These data suggest that HIV may be evolving into a commensal organism just as simian immunodeficiency virus has done in some types of monkeys. The gp120 envelope protein, Nef protein and Pol protein are particularly similar to host TCR, camouflaging HIV from the immune system and creating serious barriers to the development of safe HIV vaccines. One consequence of HIV mimicry of host TCR is that antibodies against HIV proteins have a significant probability of recognizing the corresponding TCR as antigenic targets, explaining the widespread observation of lymphocytotoxic autoantibodies in acquired immunodeficiency syndrome (AIDS). Quantitative enzyme-linked immunoadsorption assays (ELISA) demonstrated that every HIV antibody tested recognized at least one of twelve TCR, and as many as seven, with a binding constant in the 10^−8^ to 10^−9^ m range. HIV immunity also affects microbiome tolerance in ways that correlate with susceptibility to specific opportunistic infections.

## 1. Introduction

This study explores the nature of the immune response to human immunodeficiency virus infection (HIV) in light of the concepts of holoimmunity and autoholoimmunity. In particular, the mimicry of HIV for human T cell receptors (TCR) is explored; its consequences for understanding the antilymphocyte autoimmunity associated with acquired immunodeficiency syndrome (AIDS) examined; and the difficulties that it poses for vaccine development considered.

Recent research strongly suggests that organisms are not composed merely of their genetically encoded components, but develop and evolve as commensal and mutualistic super-organisms, or holobionts, that include the entire microbiome associated with the host [1,2]. Root-Bernstein [3] recently introduced the concept of holoimmunity to describe the commensal relationship between the microbiome and its host immune system. The fundamental principle of holoimmunity is that commensal or mutualistic microbes evolve to mimic not just host antigens, as Damien proposed in 1962, but the host immune system, and particularly T cell receptor (TCR) sequences, as closely as possible so as to acquire molecular camouflage from the immune system [3,4,5,6]. Selection for the microbiome is therefore mediated by the immune system. Tolerance within the immune system of an organism is therefore developed not just to its own molecular constituents, but also the molecular constituents of its integral microbiome. The immune system determines not only what microbes are pathogenic, but also what microbes are capable of interacting with the host as commensal or symbiotic constituents making up a “holobiont”. Root-Bernstein has called the system that mediates holobiont health “holoimmunity” [3].

The concept of “holoimmunity” leads logically to the concept of “autoholoimmunity”. Just as autoimmunity can result when the immune system attacks “self” antigens, autoholoimmunity can occur when the immune system turns against both “self” and microbiome antigens simultaneously. Since microbiome composition is chosen by similarity to host proteins so as to avoid immune surveillance, every microbe within the host microbiome will mimic some set of host antigens. If an autoimmune disease is triggered against such a host antigen, then the immune system will also be likely to attack those microbiome constituents that mimic the host antigen. Such cross-reactivity of the immune response may explain why every autoimmune disease is characterized by attacks on specific components of its microbiome (reviewed in [3]). Hence the term “autoholoimmunity”, since both host and microbiome are immune system targets. Additionally, a previously unrecognized aspect of such autoimmunity and autoholoimmuniity is that the immune system itself, which mimics both the host and the microbiome, can become one of the targets of attack [3].

Human immunodeficiency virus (HIV) mimicry of host antigens has been documented since the origins of the AIDS epidemic. HIV has a variety of means to avoid activating the immune system. HIV subverts various innate immunity mechanisms by downregulating toll-like receptor 9 (TLR 9); replicating too inefficiently in dendritic cells to trigger a response; and harnessing the host cytoplasmic DNAase Three-Prime Repair Exonuclease 1 (DNase TREX1) to digest nonproductive HIV reverse transcripts [7]. In macrophages, HIV uses polyadenylation specificity factor subunit 6 and cyclophilins to cloak its replication, allowing evasion of innate immune sensors [8]. In addition, HIV can subvert the complement system thereby impeding clearance of infected cells; it directly inhibits interferon and natural killer cell activity; downregulates human leukocyte antigen (HLA) expression; and prevents proteosomal degradation processes related to virus particle clearance [9]. Finally, HIV also mimics many host proteins, essentially camouflaging itself from the immune system by cloaking itself with the same tolerance mechanisms that protect the host from autoimmunity.

It has long been known that HIV mimics a wide variety of human proteins (reviewed in: [10,11,12,13,14,15,16,17]. The human proteins mimicked by HIV include the human leucocyte antigens (HLA) [18,19,20,21,22,23,24,25,26,27,28]; CD4+ T cell antigens [24,25,29,30,31,32,33,34]; interferons and interleukins [35,36,37]; NF-κB [38]; blood coagulation proteins [26,27,28,39,40,41,42,43,44,45]; integrin [46]; the substrates for cleavage and polyadenylation specificity factor subunit 6 (CPSF6) and cyclophilins (Nup358 and CypA) [8]; Hrs protein [47]; astrocyte proteins [48,49,50,51,52]; as well as a highly conserved, but unidentified, human antigen [4,5,6,53]. While exploring TCR similarities to various human and microbial proteins, it became apparent that HIV also mimics the hypervariable antigen recognition region of most human TCR.

The ability of the immune system to respond to a wide range of antigens depends on a process of recombination between diverse genetically encoded sequences and the generation of highly variable linkers that join these encoded sequences. In T cells, which implement cellular immunity and mediate B cell activation, these genetically encoded regions are made up of CD3 V-β and J-β sequences joined by D (diversity) linkers [54]. Each CD3 V-β-D-J-β sequence is presumed to have a high degree of specificity for a small number of MHC-restricted antigens. These TCR sequences are often subject to analysis with regard to their selective amplification in particular diseases. The assumption behind such studies is that CD3 V-β-D-J-β sequences are disease-specific and can reveal important information about antigen restriction.

Recent analysis of CD3 V-β-D-J-β regions of T cell receptors (TCR) from human beings has yielded the surprising observation that TCR sequences always mimic several (and sometimes many) other human proteins [3]. A further observation is that microbes that co-exist with the host as symbiotic, commensal, chronic or latent infections are much more likely to mimic TCR V-β-D-J-β regions than are acutely infectious microbes. In other words, microbes to which the immune system is tolerant are much more likely to mimic both host antigens and host TCR than are pathogens [3,4,5,6].

Selecting for a repertoire of TCR and B cell receptors (BCR) that mimic the protein sequences of the host leads to important implications for understanding how microbiome constituents are selected. TCR that are highly reactive to their host are eliminated during development leaving TCR that mimic (and therefore are non-reactive with) host antigens. The immune system therefore represents a simplified “body double” of the entire range of host antigens. Thus, the immune system can intercept and “interrogate” potentially adverse insults to the body by presenting and amplifying TCR and BCR that can prevent these insults from interacting with other host proteins. Any microbe or toxin that can interact with this “body double” has a high probability of also interacting (potentially catastrophically) with the host and therefore needs to be neutralized before it does so. Conversely, organisms and nutrients that can be beneficial to the host need to be composed of materials that either mimic the immunological “body double” or are invisible to it by not interacting with it at all. The microbiome co-exists with its host by camouflaging its components through mimicry of both host and immune system “self” (holoimmunity) [3]. Unfortunately, this molecular camouflage can produce unwanted effects as a result of autoimmune reactions. Autoantibodies can attack not just host antigens, but also the microbial mimics of these host antigens; and conversely, an immunological attack on microbiome constituents raises the risk of cross-reactivity against host mimics. Thus, every autoimmune disease results in the development of lack of tolerance to specific microbiome components, or autoholoimmunity (reviewed in [3]).

The principle that immunological “self” is encoded within TCR and BCR sequences provides a basis for analyzing the degree to which any particular microbe or antigen is likely to be able to “hide” or “camouflage” itself from the immune system by mimicking these determinants of “self” [3,4,5,6]. In particular, it is demonstrated here that HIV has evolved to mimic the T cell receptor repertoire of its host in order to avoid immune surveillance. Because of HIV mimicry of immune system antigens, if the immune system is activated to attack HIV, it has a high likelihood of producing immune responses that target not just HIV, but also TCR. Thus, HIV mimicry of TCR creates a high probability of inducing antilymphocyte autoimmunity in the host and poses significant difficulties for HIV vaccine development.

## 2. Results

Overall, the results of this study demonstrate that HIV mimics human TCR to a higher degree than any other microbe thus far examined, including commensal, mutualistic and symbiotic organisms, chronic infectious agents, and common human pathogens. This mimicry translates into anti-HIV antibodies that have a high probability of recognizing human TCR sequences with affinities associated with active autoimmune disease, at least under in vitro conditions.

**Figure 1:**
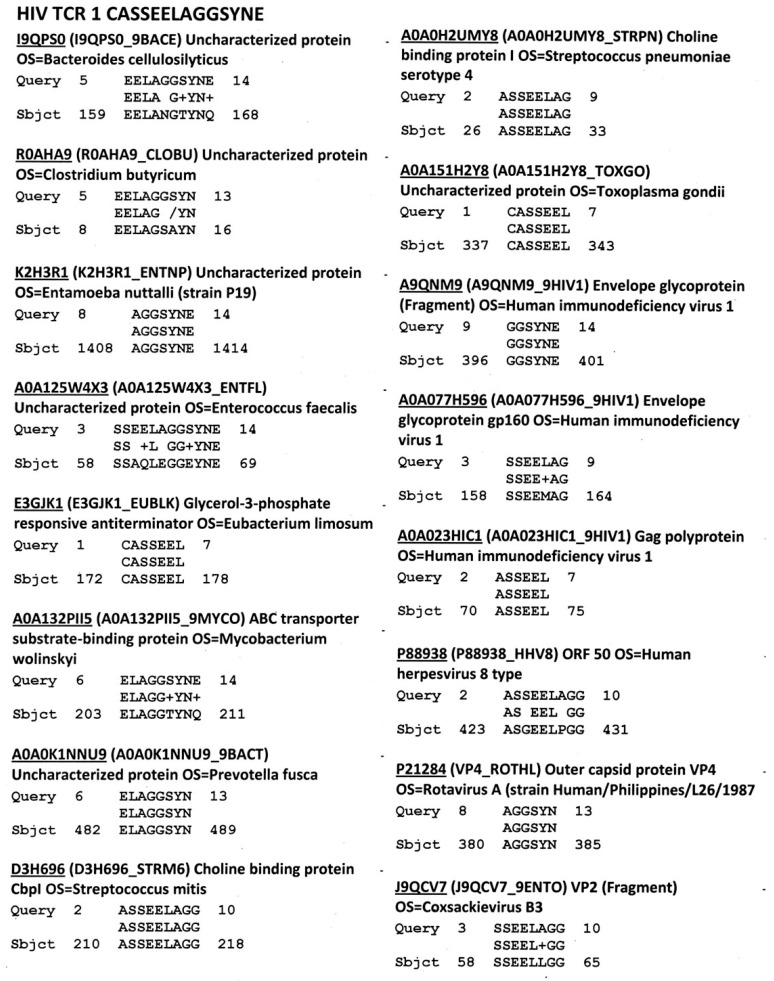
Typical (but partial) selection of HIV TCR-Microbiome protein similarities, including similarities to HIV itself (BLAST 2.0 search, UniProt protein database and virus database, *E* = 1000, no gaps). Accession numbers are for the UniProt protein database (www.expasy.org).

Figure 1 provides an example of the data generated for each TCR showing that there is a high degree of similarity between human TCR and multiple viral, bacterial and protozoal proteins. Similar data were generated for all of the 520 normal and patient TCR used in the study, the 109 HIV-associated TCR sequences, and the 201 control TCR sequences. These data were then used to generate Table 1 and Table 2, which summarize the aggregated results in terms of the percent of TCR mimicking each of the listed microbes. Statistically significant differences in these percentages are provided.

Table 1 displays two phenomena. First HIV mimics randomly selected human TCR at an unexpectedly high rate compared to any other common human viruses (average 71% as compared with the next highest mimicry rates, displayed by hepatitis C virus, cytomegalovirus and influenza A virus, each of which mimic human TCR only about 20% of the time). This rate was significantly higher than randomized TCR controls (60%) and it is highly and significantly enriched among TCR derived from HIV-infected people (87%).

Secondly, Table 1 also shows that the percentage of similarities between HIV-derived TCR and proteins of other HIV-associated viral infections such as hepatitis B, hepatitis C, Epstein-Barr virus and cytomegalovirus are also significantly increased, while no such increases are seen among viruses that are not associated as HIV cofactors in AIDS.

Table 2 also illustrates two phenomena. First, comparing Table 1 with Table 2 demonstrates that HIV mimics human TCR at a higher rate than any other class of microbes, including human commensal bacteria such as the bifidobacteria, clostridia, and lactobacilli. This observation confirms the data in Table 1 showing similarly that HIV mimics human TCR at a far higher rate than any microbe known to infect human beings.

Table 2 also illustrates the fact that TCR derived from people infected with HIV have increased similarity to a variety of bacteria and protozoal infections associated with AIDS. Significant increases in similarity compared with non-HIV populations include: corynebacteria, *Crytpococcus neoformans*, Enterobacteria, Enterococci, *Escherichia coli*, Giardia, Mycobacteria, Neisseria, Staphylococci, Streptococci, *Toxoplasma gondii*, *Trichomonas vaginalis*, and *Trypanosoma cruzi*. As with viruses, TCR similarity to bacteria and protozoal infections unassociated with AIDS are not significantly modified, or decrease in prevalence.

**Figure 2:**
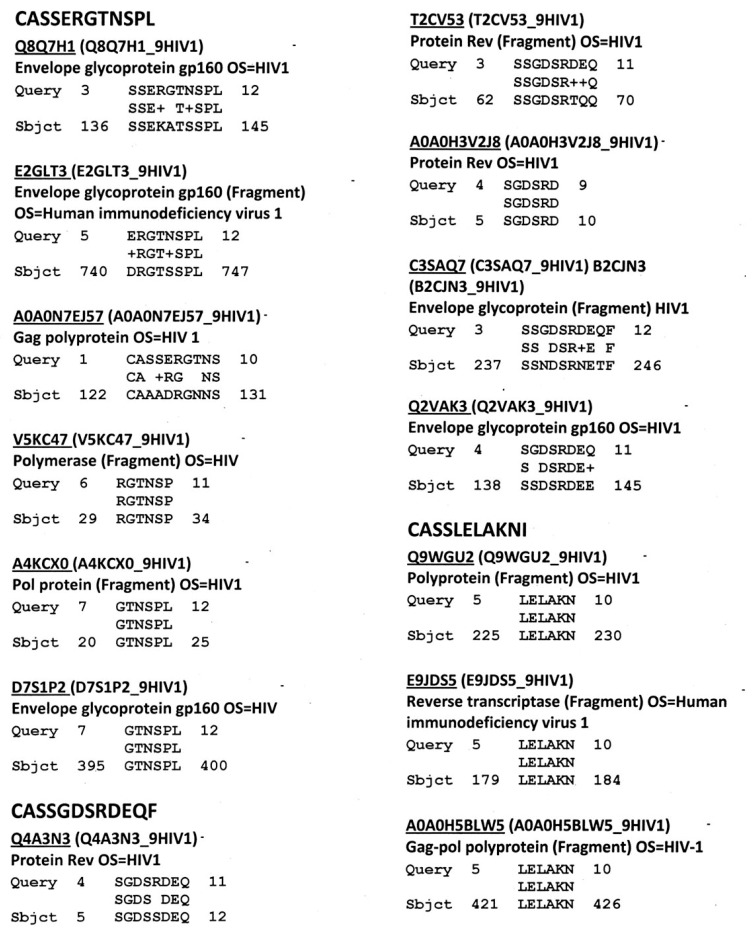
Typical selection of HIV TCR-HIV protein similarities (BLAST 2.0 search, virus database, *E* = 1000, no gaps). Accession numbers are for the UniProt protein database (www.expasy.org). See Table 3 for aggregate data for all 600 TCR examined.

Figure 2 provides representative data showing similarities between HIV TCR and HIV proteins. Rates of similarity were determined for each of the essential HIV proteins and these rates were used to generate Table 3. Figure 2 also shows that multiple HIV proteins often mimic any given TCR sequence.

Table 3 summarizes data demonstrating that the TCR mimicry encompasses all major HIV antigens, although at very different rates. HIV envelope protein (gp160) is the most likely to mimic host TCR, which is in keeping with the very high rate of mutation of gp160. A few HIV proteins, such as Vif, Vpu and Vpx rarely show similarity to human TCR.

**Figure 3:**
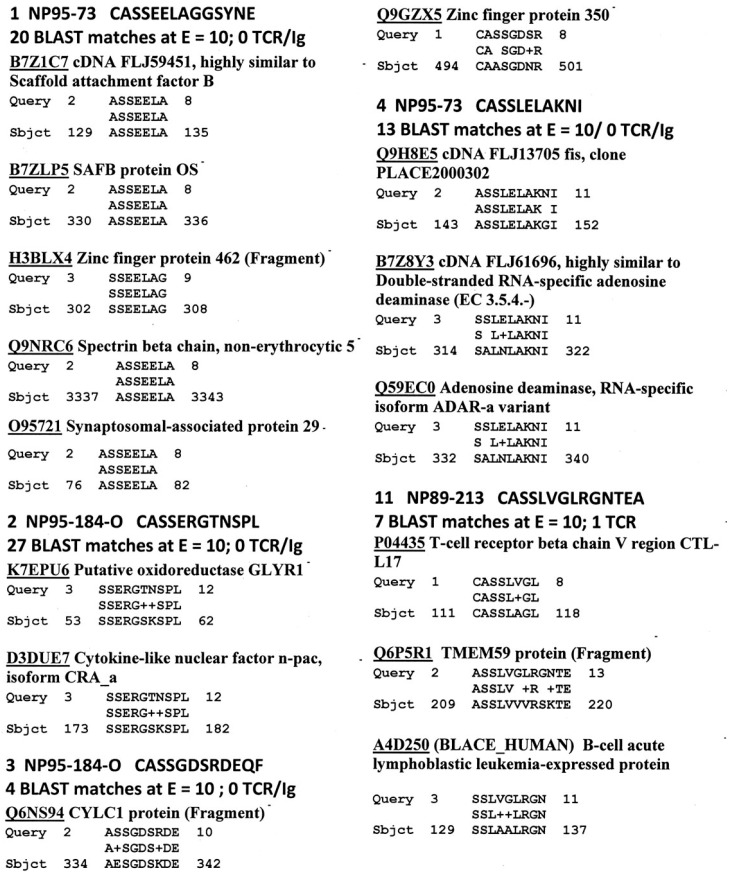
Selected examples of HIV TCR-Human protein similarities (BLAST search, *E* = 10, no gaps). See Figure 6 for examples. Accession numbers refer to the UniProt protein database (www.expasy.org).

Figure 3 provides representative (but highly selected) data demonstrating that HIV TCR mimic human proteins with great fidelity. Human protein similarities were previously reported for all of the other human TCR utilized in this study [3]. In this instance, every HIV TCR mimicked at least one, and sometimes several dozen, human proteins. Some of these human mimics include other TCR and a variety of immunoglobulins. These immune system mimics accounted for about a third of the similarities found (see Table 4).

**Figure 4:**
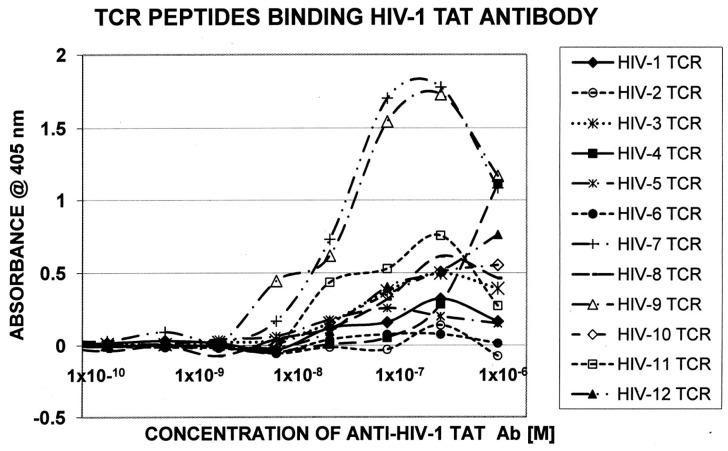
Quantitative ELISA experiments between rabbit anti-HIV-1 Tat antibody and the TCR sequences listed in Figure 2.

**Figure 5:**
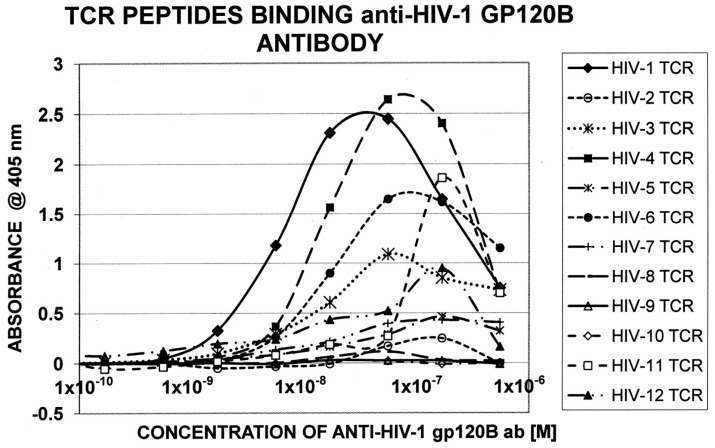
Quantitative ELISA experiments between goat anti-HIV-1 gp120B antibody and the TCR sequences listed in Figure 2.

Table 5 displays the sequences of HIV TCR that were synthesized and used to explore whether these were similar enough to HIV itself to permit recognition by antibodies against specific HIV proteins. Table 6 summarizes the results of the quantitative ELISA experiments using the TCR sequences from Table 5; representative data from these experiments are shown in Figure 4 and Figure 5. Half of the TCR-HIV antibody combinations yielded measurable binding constants ranging between 1 × 10^−7^ to 2 × 10^−9^ M. The rest of the combinations were deemed to be negative, since binding constants were either greater than 1 × 10^−6^ or no evidence of binding was obtained. (For reference, the association constant of insulin antibody for insulin, which is a primary cause of type 1 diabetes mellitus, is about 1 × 10^−8^ [55,56], whereas healthy human beings and cats have naturally circulating anti-insulin binding that binds in the range of 1 × 10^−5^ and has no clinical effect [57]). Rabbit anti-M. tuberculosis antiserum also bound measurably to some of the TCR, but a guinea-pig anti-mycobacterial antiserum did not, suggesting some specificity in both the TCR and antibody involved in the binding combinations. No significant binding was observed between cytomegalovirus or hepatitis C virus antibodies and the TCR tested here.

The main limitation of these Results is that the ELISA results that TCR peptides can bind to anti-HIV antibodies are not enough to conclude that these antibodies really recognize and bind to TCR in physiological situations. It remains to be determined whether HIV antibodies (particularly antilymphocytic autoantibodies) from people with AIDS actually bind to TCR/CD3 complexes expressed on T cells. Evidence that T cells are targeted in AIDS will be presented in the Discussion below, as will evidence that anti-HIV antibodies target specific targets in the microbiome.

### A Summary Model for HIV-Host-Microbiome Holoimmunity

**Figure 6:**
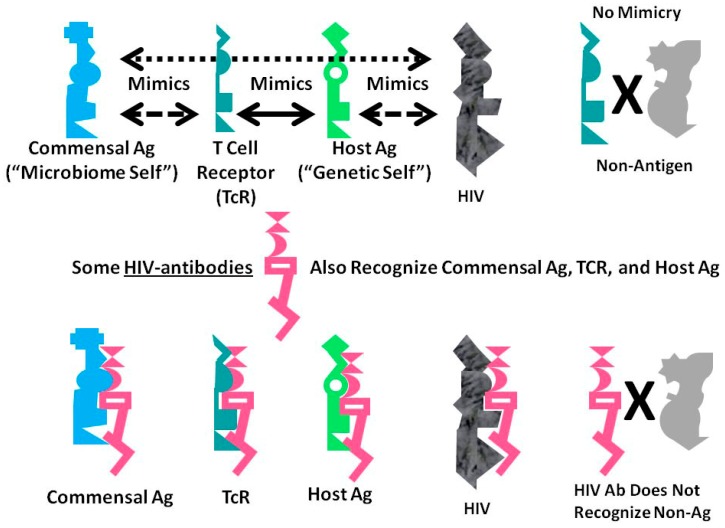
Graphical abstract of the results reported here demonstrating the range of consequences that result from the fact that HIV antigens mimic human TCR. Previous research [3,4,5,6,7] has demonstrated that human TCR mimic host proteins and the host microbiome. It has been proposed that the microbiome is selected for its compatibility with the host immune system by camouflaging itself through such molecular mimicry. This process has led to the proposal that the immune system not only mediates genetic “self”, but also the integrated holobiont “self” composed of the host and its microbiome. The data reported here strongly supports the contention that HIV mimics all components of the holobiont including, host TCR, host proteins and microbiome proteins. In order for the host immune system to eliminate HIV, it is, therefore, very likely that it will have to attack its own T cells, some of its tissues and specific components of the host microbiome. Thus, the mechanism proposed here suggests how an HIV infection, by altering specific elements of self-tolerance, may also alter tolerance for particular components of the microbiome producing some of the gut and skin symptoms commonly seen in people with AIDS. In sum, the production of lymphotcytotoxic autoantibodies in AIDS due to HIV-mimicry of host TCR should also induce autoimmunity not only against TCR, but a wider autoholoimmunity against the holobiont microbiome as well.

Figure 6 summarizes the foregoing results as an integrated mechanistic model. The immune system, in this case represented by TCR, develops to create a “body double” of the host by mimicking host antigens. Anything that can interact with this immnunological “body double” (which is to say, anything that is molecularly complementary to it) activates an immune response to block adverse interactions with the rest of the host. Through this vetting process, the host microbiome is selected for its characteristic of mimicking the host and its immunological “body double”. Such mimicry protects the microbiome constituents from immune surveillance. Thus, commensal and symbiotic constituents of the microbiome are found to mimic human TCR to a far greater degree than are pathogens. The evolution of such mimicry is probably driven by selection pressures on microbes to camouflage themselves from the immune system by looking like it, while the immune system itself has probably evolved to protect the host by mimicking key host proteins so that any microbe or toxin that can interact with these proteins will be intercepted by antibodies or TCR first.

The data presented here demonstrate that HIV mimics TCR as well as host proteins and microbiome proteins and does so to a greater degree than any other class of microbes tested other than commensal Bacteroides species. These data suggest that HIV is itself evolving into a commensal organism for its human host and helps to explain the difficulty that people actively infected with HIV have in controlling their HIV infection.

A consequence of these multiple forms of mimicry is that production of antibodies against HIV will produce antibodies that also recognize TCR (i.e., act like lymphocytotoxic autoantibodies) and that cross-react with some microbiome proteins as well. Thus, breaking tolerance to HIV will almost necessarily lead to breaking tolerance against elements “self” and the host’s microbiome. The resulting immune response will target host TCR (antilymphocyte autoimmunity) thereby reducing the ability of the immune system to respond to the pathogens such TCR would normally control. HIV mimicry of the host microbiome seems to correlate with microbial species that cause opportunistic infections in AIDS. Thus, autoimmunity against TCR initiated by HIV may result in specific “holes” in the TCR network necessary to control such infections.

## 3. Discussion

To summarize the experimental results, HIV mimics human TCR at a far higher rate than any other microbe thus far examined. In context, this finding strongly suggests that if HIV is not already a commensal organism for human beings, it is well on its way, just as simian immunodeficiency virus (SIV) is commensal for some monkey species [59,60]. The support for this conclusion is that previous research has demonstrated that the more similarities a microbe has to the TCR repertoire of a host organism, the greater the probability is that this microbe is a commensal organism or symbiont for its host [3,4,5,6,7]. Indeed, in this study, it is demonstrated that chronic and latent viruses such as hepatitis, cytomegalovirus and papilloma are far more likely to mimic host TCR sequences than are acutely infectious viruses. Commensal gut bacteria such as Bacteroides, Clostridia and Lactobacilli are the only microbes with rates of TCR similarity that are even close to that displayed by HIV. The similarity of HIV for host proteins extends significantly beyond TCR sequences as well: Lucchese et al. [61] found that at the pentapeptide level, the HIV-human proteome overlap consists of 14,227 matches disseminated throughout 10,312 human proteins. HIV, in short, is very well camouflaged from immunological surveillance at the molecular level.

The mimicry of human HIV-associated TCR for HIV itself was confirmed using quantitative ELISA. All of the polyclonal HIV antibodies tested recognized one or more human TCR, and six of the interactions were certainly strong enough to provoke an active autoimmune response and so may represent lymphocytotoxic autoantibodies. The shift from a Th1 to a Th2 immune response to HIV that often signals a transition to the development of AIDS may therefore require overriding tolerance mechanisms and thus, necessarily induces autoimmunity. Cytomegalovirus, hepatitis C and tuberculosis antibodies did not show either as broad or as high-affinity interactions with the same TCR indicating that the HIV mimicry of human TCR is relatively unique to this virus.

The data also demonstrate that all TCR are strongly selected to mimic the human proteome itself [3,4]. Indeed, approximately 15 to 20 percent of TCR sequences are widely shared among diverse individuals as “public” sequences [62,63,64] so that the actual proteins mimicked by TCR are probably highly conserved from one individual to another. Williams et al. [65] have demonstrated, for example, that anti-HIV gp41 antibodies recognize a range of human proteins, and that the interaction between these antibodies and host proteins prevents neutralization of HIV by adsorbing significant amounts of antibody.

The potential for autoimmunity following HIV antibody production may extend beyond TCR as targets. Rolland et al. [66] have demonstrated experimentally that T cell recognition of HIV antigens is inversely related to the degree to which they mimic human proteins in general: the more similar an HIV antigen is to a human antigen, the more likely it will be tolerated as “self”. In light of recent observations that TCR mediate tolerance between both the human proteome and the microbiome by means of just such similarities [3,4,5,6], it seems likely that HIV has evolved very effectively to camouflage itself by mimicking not only a wide range of human proteins, but also, through its mimicry of both HLA and TCR proteins, the human immune system itself. These results are also consistent with HIV being closely related to class K human endogenous retroviruses [63,64,65,66,67,68,69]. Additionally, this hypothesis is also consistent with SIV being non-pathogenic in some monkey species due a T-cell-mediated mechanism [59,60]. In short, HIV may have evolved, or is in the process of evolving, into a commensal organism that is only pathogenic under extraordinary conditions of immunological dysfunction.

An important consequence of the concept of holoimmunity is that the same immunological mechanisms that determine “self”-“nonself” also operate to regulate what microbes can participate as non-pathogenic components of the host microbiome. Microbiome microbes mimic host antigens. Thus, HIV mimicry of its host will, according to holoimmunity, also result in mimicry of microbiome constituents. This appears to be the case. Notably, TCR mimicking HIV also mimic particular components of the human microbiome, in particular those microbes most associated with AIDS-related infections. This result suggests that the shift in HIV-related TCR shifts the immune system’s tolerance (or intolerance) for the diseases that develop during AIDS, especially the opportunistic gut infections that occur in many people with AIDS such as pathogenic Chlostridia, Enterobacteria, Enterococci, and Giardia. TCR mimicry also significantly increases to other microbes such as Mycobacteria, Staphylococci, Streptococci, and Trichomonas, which are common concomitants of AIDS. The increase of TCR mimicry of these microbes suggests an attempt by the immune system to mediate disease, as well as selection by the microbiome during AIDS development toward microbes that increasingly avoid immune detection through mimicry [3,4,5,6]. Williams et al. [65] and Trama et al. [70] have, indeed, reported significant cross-reactivity between antibodies against HIV and commensal bacteria as well as diversion of HIV-vaccine-related antibodies from HIV to these microbiome microbes. More specifically, they found that 82% of anti-HIV gp41 antibodies cross-reacted specifically with commensal gut bacteria, just as would be expected from the TCR data provided here.

An unusual prediction that follows from mutual HIV-TCR-host-microbiome mimicry is that risk of HIV infection may be mediated by the microbiome itself. One of the holoimmunity principles stated by Root-Bernstein [3] is that, because the microbiome has evolved to mimic the host and more specifically host TCR, the microbiome functions in part as a secondary immune system that can recognize and defend against the same antigens that are recognized by TCR. A healthy microbiome should therefore be a robust defense against HIV infection. One of the best-characterized instances of such a protective effect is seen in the relationship between vaginal microbiome health and risk of HIV acquisition in women. A healthy vaginal microbiome, dominated by normal Lactobacilli, reduces HIV and sexually transmitted infections among African women [71,72], while vaginal dysbiosis results in production of HIV-enhancing factors and increased HIV risk [73,74]. Prophylactic use of Lactobacilli introduced into the vagina has resulted in lowered risk of HIV and herpes simplex virus acquisition in women with dysbiosis [75,76]. The results of HIV-TCR-microbiome similarities found here, however, taken in conjunction with the results of Trama et al. [70], Petrova et al. [77], and Williams et al. [65] concerning HIV cross-reactivity with many commensal microbes, suggest that microbiome-HIV interactions are mediated by many microbes in addition to Lactobacilli. Thus, a broader microbiome approach may be required to effectively mediate HIV infection.

These results have important implications for understanding HIV pathogenesis even beyond how the microbiome mediates HIV infection and what co-infections the immune system can recognize as HIV infection progresses. One additional implication is that anti-HIV and anti-lymphocyte autoimmunity are integrally related aspects of AIDS [10,11,12,13,14,15,16,17,18,19,20,26,27,28,29,30,31,32,33,34,78,79,80,81]. Indeed, Kion and Hoffman demonstrated that it is possible to produce an animal model of AIDS using lymphocytes as antigens; the resulting autoimmune process not only destroys T cells but is also cross-reactive with HIV [80,81]. These results can be explained by the very striking antigenic similarities between TCR and HIV revealed here, especially if one considers the Hoffmann-Kion model as a form of autoholoimmunity that can be initiated by either host or microbial mimics.

Another important implication of the data reported here is to explain the almost universal finding of anti-T cell autoimmunity in people with AIDS [15,16,26,29,30,31,32,33,34]. The switch from Th1 to Th2 immunity that characterizes the development of HIV into AIDS is almost always accompanied by the production of lymphocytotoxic autoantibodies (LCTA). In light of the mimicry of HIV for TCR, LCTA would be an almost unavoidable consequence of developing active Th2-mediated immunity to HIV as almost any anti-HIV antibody will cross-react with some subset of TCR. Antibody against HIV will therefore induce T cell destruction as a by-product, making autoimmunity an integral part of AIDS pathogenesis [15,16]. One consequence of this HIV-induced intra-system autoimmunity is inflammation within the lymphatic system itself, leading to deposition of fibrotic tissue. This inflammatory process is inversely proportional to T cell count [82]. Thus, once again, AIDS can be considered a form of autoholoimmunity.

There are also treatment implications of the mimicry of HIV for TCR. First, given that about seventy percent of randomly selected TCR sequences in normal human beings, and up to ninety percent in HIV-infected individuals mimic HIV proteins, it is extremely unlikely that it will be possible to produce a whole-virus HIV vaccine that does not mimic host TCR to a very significant degree. Such vaccines will either be treated immunologically as “self” and therefore fail to induce immunity or, if the immune system can be tricked into being activated, the response will cross-react with one or more subsets of TCR, therefore causing the very T cell destruction that the vaccine is intended to avoid. The repeated failure of HIV vaccines, and particularly of gp160 (envelope protein)-based vaccines (which have the highest similarity to TCR) is a matter of record [83,84,85]. In addition, the induction of anti-T cell autoimmunity has, been reported in some failed trials, with concomitant enhancement of HIV acquisition [86,87,88]. In fact, components of the envelope protein are actively tolerized by the human immune system [89] so that HIV-infected patients who do develop broadly neutralizing antibodies that can target multiple HIV strains also produce high levels of autoantibodies and low levels of regulatory T cells [90]. All of these results follow logically from the observation of HIV-TCR-human proteome mimicry reported here.

Alternative approaches to developing HIV vaccines, such as Gag/Pol/Nef vaccines, have also failed [91], perhaps because these antigens are not readily recognized by antibodies, but also perhaps because the target proteins, like envelope protein-based vaccines, also mimic host antigens and TCR sequences. The data presented here suggest that the best components from which to construct an HIV vaccine that would be recognized as clearly “non-self” and have minimal cross-reactivity with human lymphocytes will be those proteins that have the least similarity to host TCR, which means the HIV regulatory proteins Tat, Rev, Vif, Vpu and Vpx (Table 4 and Table 5). Unfortunately, these proteins are not displayed in the native virus, are generally inaccessible to antibody, and are therefore likely to be ineffective as components of vaccines.

In light of HIV mimicry of both HLA and TCR, rather than attempting to vaccinate against HIV directly, some investigators have argued that alloimmunization appears may be more effective approach to treating existing HIV infection to reestablish tolerance [83,92,93]. Another possibility might be to utilize a virus sufficiently similar to HIV to cross-react with it, but sufficiently unadapted to human beings to be recognized as “non-self” and therefore to activate a robust antibody response that does not cross-react with TCR. One such possibility might be simian immunodeficiency virus (SIV) [94], which did not show up as a TCR mimic in the homology searches conducted here.

## 4. Methods

### 4.1. Proteonomics

The results reported here are an unexpected product of a study of TCR mimicry related to tolerance for the human microbiome [3] That study employing 520 published CD3 V-β-D-J-β regions from peripheral blood human TCR derived from normal control patients, people with various monoinfections (such as influenza, HTLV, *Streptococcus* type A, and tuberculosis), and the antisense versions of these monoinfection TCR. In addition, TCR from people with two autoimmune diseases (Crohn’s and type 1 diabetes mellitus) were explored for similarities to human self-proteins and microbiome antigens [references and all sequences available in [3]. An additional 109 TCR sequenced from people with AIDS were added to the present study (see Supplementary Material). These TCR were acquired from the following sources: [58,95,96,97]. Finally, two sets of control TCR were utilized in this study. The first was a set of 101 “antisense TCR” sequences generated from 101 normal patient control TCR by using theses sequences to predict their complementary or “antisense” sequences (see [3] for details and sequences). An additional 100 random TCR-like sequences of 15 amino acids in length (the average length of the TCR used in this study) were generated using a random peptide sequence generator (http://web.expasy.org/randseq/)—see Supplementary Material for sequences. The antisense and random TCR results were aggregated to provide a robust control of 201 variously randomized TCR-like sequences with which to compare the patient-selected TCR results.

Similarity searching of proteonomic databases provided the probabilities that any given TCR would be mimicked by a protein in any given species or genera of microbes. Data on mimicry was obtained by using each TCR sequence as a search string in a BLAST 2.0 search (www.expasy.org) with the *E* value set to 1000 with 1000 sequences displayed and the gapped sequence feature turned off. The results were hand-curated for human pathogens, commensal and symbiotic microbes after separate searches of the entire UniProtKB, bacterial and viral databases. Mimicry was defined, for the purposes of this study, as having at least six identical amino acids in a sequence of ten. This criterion was employed because various studies have demonstrated that this degree of similarity is often sufficient to result in antibody cross-reactivity between peptides [98,99,100,101]. Moreover, similar criteria have been used in previously published HIV-mimicry studies (e.g., [23,61,83]. For example, Lucchese et al. [61], compared the amino acid primary sequence of HIV-1, isolate CDC-451 with the human proteome and found that HIV-1 shares 50 heptapeptides and three octapeptides with the human proteome, of which 34 are experimentally validated epitopes targeted by immune responses following HIV-1 infection. It has previously been demonstrated that the results are not significantly skewed by the number of taxons or specific protein entries in the UniProtKB database [3].

A similar BLAST 2.0 (www.expasy.org) search of the human proteome was conducted with the HIV TCR with the BLOSSUM80 algorithm, *E* = 10, no gaps and the 100 best matches displayed.

### 4.2. Statistics

A χ-squared test (http://www.quantpsy.org/chisq/chisq.htm) was used to compare the observed frequencies of the microbial similarities to TCR. Because multiple χ-squared tests were run on the same sets of data, a Bonferroni correction was employed (http://www.winsteps.com/winman/bonferroni.htm). With about 40 microbes in both the bacterial/protozoal list and the virus list, the Bonferroni correction to achieve the equivalent of *p* < 0.5 significance was reset to *p* < 0.001. Only results that met or exceeded *p* < 0.001 significance are therefore reported.

### 4.3. T Cell Receptor Peptide Synthesis

A random selection of twelve TCR sequences from the HIV-positive patients (Figure 2 [58]) was synthesized by the Macromolecular Synthesis and Mass Spectrometry Facility of the Biochemistry Department of Michigan State University. These were purified using HPLC to at least 98% purity as determined by mass spectrometry.

### 4.4. Antibodies

Guinea pig anti-mycobacterium and Rabbit anti-M. tuberculosis (Biodesign, Kennebunkport, ME, USA). The following antibodies were obtained from the NIH AIDS Reagent Program, Division of AIDS, NIAID, NIH: Goat anti HIV-1 gp120 (PB1) (NIH 36) with specificity for IIIB gp120 (aa 295–474); Goat anti HIV-1 gp120 (PB1) (NIH 41) with specificity for MN and RF gp 120 (aa 295–474); Sheep anti HIV-1 p17 (NIH 286); Sheep anti HIV-1 p24 (NIH 287); Sheep anti-HIV-1 gp120 (NIH 288); Rabbit anti HIV-1 Tat (NIH 705); Mouse anti-CMV (AD 169) gB (NIH 1592); Rabbit anti HIV-1 Nef (NIH 2949); Rabbit anit HIV-1 Protease (NIH 4105); Rabbit anti HIV-1 HIV-1 p17 (NIH 4811); Rabbit anti-HIV-1 RT (NIH 6195). NIH 36 and 41 were obtained through the NIH AIDS Reagent Program, Division of AIDS, NIAID, NIH [102]. NIH 286, 287, 288 were obtained through the NIH AIDS Reagent Program, Division of AIDS, NIAID, NIH: Antiserum to HIV-1 p17 from Michael Phelan. NIH 705 was obtained through the NIH AIDS Reagent Program, Division of AIDS, NIAID, NIH: Antiserum to HIV-1 Tat from Bryan Cullen [103]. NIH 1592 was obtained through the NIH AIDS Reagent Program, Division of AIDS, NIAID, NIH: Monoclonal Antibody to CMV (AD169) gB from Lucy Rasmussen [104]. NIH 2949 was obtained through the NIH AIDS Reagent Program, Division of AIDS, NIAID, NIH: Catalog #2949, Anti-HIV-1 Nef Polyclonal from Ronald Swanstrom [105]. NIH 4105 was obtained through the NIH AIDS Reagent Program, Division of AIDS, NIAID, NIH: Anti-HIV-1 Protease Polyclonal. NIH 4811 was obtained through the NIH AIDS Reagent Program, Division of AIDS, NIAID, NIH: Anti-HIV-1 p17 Polyclonal from Paul Spearman and Lingmei Ding. NIH 6195 was obtained through the NIH AIDS Reagent Program, Division of AIDS, NIAID, NIH.

### 4.5. ELISA

Quantitative enzyme-linked immunoadsorption assays (ELISA) were run on the TCR peptides listed in Figure 1 against the antibodies listed above. Briefly, ten serial dilutions by thirds of a 1 mg/mL solution of each TCR were made in pH 7.4 phosphate buffer. 100 µL of each dilution was added to each well in a 96-well Costar 3590 high affinity ELISA plate and run in triplicate. The plate was incubated for one hour at room temperature and then washed three times with 2% TWEEN 20 in phosphate buffer. Next, 200 µL of a 1% polyvinylalcohol solution was added as a plate blocker to each well, incubated for 1 hour, and then washed as above. 100 µL of 1:1000 primary antibody was added to each well, incubated and washed. The appropriate secondary HRP-labeled antibody (1:1000) was then added to each well, incubated and washed. Finally, 100 µL of ABTS single reagent was added to each well, incubated for 30 minutes, and then read at 405 nm in a SpectraMax UV-Vis scanning spectrophotometer. Data were analyzed and plotted using Excel. Most of the ELISAs were run at least twice for confirmation. Only those binding curves that exceeded an absorbance value equal to or greater than 0.5 O.D. at 405 nm were calculated; anything below this absorbance cut-off was assumed to be non-specific binding.

## 5. Conclusions

In sum, it has been well established by previous investigators that HIV mimics many human proteins and in particular HLA, while here it is demonstrated that HIV also mimics TCR and this mimicry translates into antibody specificity for about half of the TCR that display such mimicry. Thus, an active antibody response to HIV may result in lymphocytotoxic autoantibodies directed at TCR. The immune system faced with an HIV infection is therefore faced with a difficult “decision”: it may ignore the infection, risking overwhelming infection, or it may respond to the infection, potentially inducing an autoimmune response. Since immune activation requires presentation of antigen by HLA to an appropriate TCR in the presence of CD4, and HIV mimics two of the three key molecules in this process (HLA and TCR), its camouflage is doubly subversive, which may help to explain the difficulty in mounting an effective immune response to HIV.

I would like to speculate further that HIV is either already commensal in human beings or well on the way to becoming so. The import of this speculation is that HIV may not, intrinsically, be pathogenic just as other commensal organisms are not ordinarily pathogenic. In this context, it is advisable to remember that HIV is very difficult for a healthy human being to acquire. Documented rates of acquisition of HIV among healthcare providers exposed subcutaneously to HIV are in the order of 1 in 3000 exposures (which compares to 1 in 2 for hepatitis B) [106]. Healthy heterosexuals engaged in vaginal intercourse with an HIV-positive partner [107], have very low rates of seroconversion (1 in 3000 to 1 in 8000). HIV appears to require already impaired immunity or various cofactors to overcome immunological tolerance to it in order to induce active disease [15,81,108,109,110,111]. Such pre-conditions would be consistent with HIV being a commensal, or near-commensal, organism for human beings.

Other important implications also follow from the observation that HIV mimics TCR. In order to control an HIV infection, the immune system must break “self” tolerance. An unavoidable consequence of breaking self-tolerance is autoimmunity directed at the immune system itself, which may help to explain why anti-retroviral therapies have not proven sufficient to cure AIDS. Such autoimmunity can also be expected to ensue as a result of active vaccination against HIV making the development of any HIV vaccine highly problematic.

Equally importantly, the results described here suggest that an active response to HIV will also spill over to the host’s microbiome, shifting tolerance for some of its components away from normal, and opening up the host to opportunistic infections. Conversely, a healthy microbiome may assist in preventing HIV acquisition and transmission. Thus, within the concept of the human being as a holobiont, the trifecta of TCR-host-microbiome similarities that is manipulated by HIV mimicry of all three results in systems-wide attacks following active infection that are best described by the concept of autoholoimmunity.

## Figures and Tables

**Table 1:** HIV mimics TCR far more frequently than any other virus.

Human Virus	TCR AVG% *N* = 386	Chi 2 *p* Val HIV vs. AVG	HIV TCR% *N* = 109	Chi 2 *p* Val HIV vs. Rand	Rand + Anti TCR% *N* = 201	Chi 2 *p* Val AVG vs. Rand
**Adenovirus**	**14**		**16**		**15.5**	
Astrovirus	1		2		2.5	
Bocavirus	0.5		2		4	
Cardiovirus	0.5		0		1.5	
Coronavirus	5		9		4	
Coxsackie A	5	0.001	12	1.3 × 10^−7^	3	
Coxsackie B	3.3		4		0	
**CMV**	**22.5**		**30**	**3.1 × 10^−6^**	**13.5**	
Echoviruses	7.2		6		2.5	
Enteroviruses	7.3		14	3.3 × 10^−7^	4	
EBV	8.1	0.003	16	4.1 × 10^−6^	5.5	
HAV	0.8		4		0	
HBV	7	1.6 × 10^−5^	18		21.5	1 × 10^−8^
**HCV**	**21.5**	**7.3 × 10^−5^**	**41**		**33**	
HEV	2.7		5		14	0.001
HHV1	4.5		7		14.5	0.004
HHV2	4.5	0.001	11		7.5	
HHV6	3.7	8.4 × 10^−7^	13		8.5	
HHV8	3.7		8		8.5	
**HIV-1**	**71.2**	**2.6 × 10^−6^**	**87**	**6 × 10^−8^**	**60.5**	
HTLV I and II	2.7		2		6	
**Infl A Virus**	**20.8**		**27**		**29**	
Infl B virus	1.3		1		5.5	
Infl C virus	0.7		1		1	
Jap enc virus	1.5		3		0.5	
Measles virus	2.8		2		8	
Mumps virus	0.5		0		3	
Norovirus	5.3		8		5.5	
**Papilloma virus**	**15.5**	**0.003**	**29**		**33.5**	**0.0001**
Parainfluenza	0.7		4		3	
Polio virus	0.5		4		1	
Polyoma virus	2		1		1.5	
Reovirus	2.3		3		2.5	
RSV	0.5		2		7.5	0.007
Rhinovirus	4		9		5.5	
Rotaviruses	6.3	1 × 10^−5^	17		14.5	0.0007
Rubella	1.3		2		4	
Varicella zoster	4.3		6		4	

Frequency of virus proteins mimicking T cell receptors (TCR) from a variety of patient populations: TCR AVG is the average derived from (Root-Bernstein, 2016 [3]) and consists of the sum of TCR matches from NOR = normal (no infection) controls; CONT = people with mono-infections; CD = Crohn’s disease; T1DM = diabetes; HIV = people with human immunodeficiency virus infections; RAND & ANTI are hypothetical TCR generated by two methods, a random peptide generator (100 sequences) or using the antisense sequence predicted from the normal controls (101 sequences). Bolded material indicates the viruses that mimic TCR on consistently more than 10% of the time. Statistics are reported only for significant differences (*p* < 0.001 by χ squared with Bonferroni correction for the multiple viruses tested).

**Table 2:** HIV mimics TCR far more frequently than any bacterium, fungus or protozoa with the exception of the class of Bacteroides species.

Human Microbe	AVG TCR% *N* = 386	Chi Sq *p* Val AVG vs. HIV	HIV TCR% *N* = 109	Chi Sq *p* Val Rand vs. HIV	Rand + Anti TCR % *N* = 201	Chi Sq *p* Val AVG vs. Rand
*Bacillus cereus*	32.0		37		42.5	
***Bacteroides spp.***	**64.0**		**70**		**74**	
***Bifidobacteria***	**27.5**	**0.005**	**40**		**42.5**	**0.0008**
*Bordetella pertussis*	5.0		9		16.5	0.002
*Campylobacter jejuni*	4.8		5		6	
*Candida albicans*	3.9	3 × 10^−6^	13		10	0.0002
*ardiobacteria*	0.5	7 × 10^−6^	4		8	<1 × 10^−10^
*Chlamydia*	5.3	<1 × 10^−10^	26	<1 × 10^−10^	1	
***Clostridium spp.***	**50.5**	**0.007**	**64**		**61.5**	
***Coccidiodes spp.***	**39.5**		**33**		**26**	
*Coprococcus*	10.5		14		7.5	
*Corynebacteria*	14.0	<1 × 10^−10^	43		53.5	<1 × 10^−10^
*Cryptococcus neoformans*	1.0	<1 × 10^−10^	24		18.5	<1 × 10^−10^
*Cryptosporidium*	15.5		13		18	
*Entamoeba*	6.7	1 × 10^−8^	21		24.5	<1 × 10^−10^
*Enterobacter spp.*	12.0	<1 × 10^−10^	46	0.002	61	<1 × 10^−10^
*Enterococcus spp.*	22.3	6.6 × 10^−7^	43	0.0002	26.5	
***Escherichia coli***	**26.8**		**36**	**0.0005**	**53.5**	**9 × 10^−8^**
*Eubacterium*	22.0	0.0007	36		47.5	3.3 × 10^−7^
*Gardnerella vaginalis*	13.5		9		11	
*Giardia*	11.5	0.0003	23		27	1.2 × 10^−6^
*Haemophilus influenzae*	4.6		6		4	
*Helicobacter pyelori*	7.6		8		7.5	
*Histoplasmosis*	0		0		1	
*Isospora*	0		0		0	
*Klebsiella pneumoniae*	10.9		17		23.5	
***Lactobacillus spp.***	**40.0**		**45**	**0.0001**	**63.5**	**1 × 10^−6^**
*Legionella pneumophila*	6.0		6		11	
*Listeria*	8.9		8	0.008	16	
*M. tuberculosis*	8.3	0.0004	18		29	5 × 10^−6^
***Atypical Mycobacterium***	**35.0**	**<1 × 10^−10^**	**74**		**76.5**	**<1 × 10^−10^**
*Mycoplasma*	9.0		8		13.5	
*Neisseria*	6.2	0.0001	22		20.3	0.0005
*Pneumocystis*	9		6		13	
***Prevotella spp.***	**47.5**		**50**		**50.5**	
*Pseudomona aeruginosa*	11.6*	0.002	21		31.5	0.0003
*Salmonella*	10.5	<1 × 10^−10^	29		29	<1 × 10^−10^
*Shigella dysenteriae*	4.0		3		5.5	
*Staphylococcus*	12.5	<1 × 10^−10^	34		27.5	0.0008
***Streptococcus spp.***	**31.0**	**<1 × 10^−10^**	**73**		**80.5**	**<1 × 10^−10^**
*Toxoplasma gondii*	25.0	3 × 10^−5^	43		38	0.003
*Treponema pallidum*	1.3		3		0	
*Trichomonas vaginalis*	15.0	2 × 10^−8^	35		33	4.6 × 10^−7^
*Trypanosoma cruzi*	14.0	<1 × 10^−10^	36		31	9.6 × 10^−7^

Frequency of non-virus microbial proteins mimicking T cell receptors (TCR) are from the same sets described in Table 1. Bolded material indicates the bacteria and protozoa that mimic TCR on average more than 30% of the time. Statistics are reported only for significant differences (*p* < 0.001 by χ squared with Bonferroni correction for the multiple microbes tested). See [3] for additional data.

**Table 3:** Frequency with which HIV TCR mimic HIV proteins. See Figure 2 for examples.

HIV-1 Proteins	% TCR Normal Mimics	% TCR Control Mimics	% TCR Antisense Control Mimics	% TCR Random Sequence Mimics	% TCR HIV Mimics
Env (envelope proteins, gp160, gp120, gp41)	70	63	27	40	69
Pol (reverse transcriptase, protease, RNAase, integrase)	30	20	22	21	20
Gag (viral capsid and matrix, p24, p17, p9, p6)	20	6	18	15	30
Nef (regulatory: negative replication factor)	11	9	17	11	0
Vif (regulatory: virion infectivity factor)	2	4	8	7	0
Vpu (regulatory: viral protein U, virus assembly)	3	3	1	6	3
Tat (regulatory: transactivator of RNA transcription)	5	2	6	4	0
Rev (regulatory: stimulates protein production)	7	2	9	6	13
Vpr (regulatory: viral protein R, protein production accelerator)	0	1	3	3	3
Total HIV mimics	148	110	111	113	145
One or more HIV mimics	79	76	61	60	80
No HIV mimics	21	24	39	40	20

**Table 4:** Frequency with which HIV TCR mimic human proteins (see Figure 3 for examples). The number of matches is provided plus or minus the standard deviation for total human protein similarities; for the subset of other TCR and immunoglobulins; and for the subset of somatic proteins.

Average Total BLAST Human Similarities per HIV TCR	Average HIV TCR Similarities to Other Human TCR/Ig per HIV TCR	Average Non-TCR/Ig Human Similarities per HIV TCR
28.5 ± 19.0	10.7 ± 10.7	17.5 ± 16.5

**Table 5:** List of TCR sequences from HIV patients synthesized for use in ELISA experiments (randomly selected from Lin et al., 2005 [58]).

TCR ID	Sequence	Patient
HIV 1	CASSEELAGGSYNE	NP95-73
HIV 2	CASSERGTNSPL	NP95-184-O
HIV 3	CASSLELAKNI	NP95-184-O
HIV 4	CASSGDSRDEQFF	NP95-73
HIV 5	CASSLWVTGGEQFF	NP89-213
HIV 6	CASSFSSGRPGELF	NP95-73
HIV 7	CASSLTVSSYNEQ	NP95-73
HIV 8	RCASSSGANV	NP95-73
HIV 9	FCASRFERELGQPQ	NP-94-34
HIV 10	LCSVVTGDGYTF	NP95-184-O
HIV 11	CASSLVGLRGNTEA	NP89-213
HIV 12	CASSLASYTEA	NP94-34

**Table 6:** Results of quantitative ELISA experiments (see Figure 4 and Figure 5 for examples of data) between HIV-antibodies and TCR from HIV-infected patients (see Table 3).

	HIV-1	HIV-2	HIV-3	HIV-4	HIV-5	HIV-6	HIV-7	HIV-8	HIV-9	HIV-10	HIV-11	HIV-12
**HIV-1 gp120** **( × 10^–6^)**	**0.0024**	>1	**0.014**	**0.016**	>1	**0.018**	>1	>1	>1	>1	**0.10**	**0.055**
**HIV-1 Pol** **( × 10^–6^)**	**0.017**	**0.012**	**0.0081**	>1	>1	>1	>1	**0.036**	>1	**0.028**	**0.025**	**0.0085**
**HIV-1 Gag** **P24 ( × 10^–6^)**	**0.052**	>1	>1	>1	>1	>1	>1	**0.078**	**0.08**	>1	>1	>1
**HIV-1 Gag** **P17 ( × 10^–6^)**	**0.022**	**0.018**	**0.010**	>1	>1	>1	>1	>1	>1	>1	>1	>1
**HIV-1 Tat** **( × 10^–6^)**	>1	>1	**0.028**	>1	>1	>1	**0.020**	**0.034**	**0.022**	>1	>1	**0.015**
**HIV-1 Nef** **( × 10^–6^)**	0.031	>1	>1	>1	**0.0037**	>1	**0.011**	**0.033**	>1	**0.024**	**0.072**	**0.0085**
**CMV (AD169) Mab ( × 10^–6^)**								>1	>1	>1		
**HCV Core** **( × 10^–6^)**	>1	>1	>1	>1	>1	>1	>1	>1	>1	>1	>1	>1
**RBT M. tuber.** **( × 10^–6^)**	**0.011**	>1	>1	>1	>1	**0.10**	>1	>1	**0.0085**	>0.1	>1	>1
**Anti-Mycob.** **( × 10^–6^)**	>1	>1	>1	>1	>1	>1	>1	>1	>1	>1	>1	>1

Binding constants highlighted in black are in the range of 10^−9^ M; highlighted in medium grey are in the range of 10^−8^ M; highlighted in light grey are in the range of 10^−7^; and those with white backgrounds had binding constants above 10^−6^ M. CMV = cytomegalovirus; HCV = hepatitis C virus; M. tuber. = Mycobacterium tuberculosis; anti-mycobacterium is a polyclonal antibody against mycobacteria species in general.

## Supplementary Materials

Supplementary materials can be found at www.mdpi.com/1422-0067/18/10/2091/s1.
