# Supplementary material for: Human Immunodeficiency Virus Proteins Mimic Human T Cell Receptors Inducing Cross-Reactive Antibodies
Source: Int J Mol Sci. 2017 Oct 3;18(10):2091. doi: 10.3390/ijms18102091 (PMC5666773; doi:10.3390/ijms18102091)
Supplement: Supplementary file 1 [file ijms-18-02091-s001.pdf]

HIV TCR list

Lin, W. L., Fincke, J.E., Sharer, L.R., Monos, D.S., Lu, S., Gaughan, J., Platsoucas, C.D., Oleszak, E.L. Oligoclonal T cells are infiltrating the brains of children with AIDS: sequence analysis reveals high proportions of identical β-chain T-cell receptor transcripts. Clinical and Experimental Immunology 2005; 141: 338-356. (4 patients; 30 sequences)

1 NP95-73 CASSEELAGGSYNE

2 NP95-184-O CASSERGTNSPL

3 NP95-184-O CASSGDSRDEQF

4 NP95-73 CASSLELAKNI

5 NP89-213 CASSLWVTGGEQFF

6 NP95-73 CASSFSSGRPGELF

7 NP95-73 CASSLTVSSYNEQ

8 NP95-73 RCASSSGANV

9 NP-94-34 FCASRFERELGQPQ

10 NP95-184-O LCSVVTGDGYTF

11 NP89-213 CASSLVGLRGNTEA

12 NP94-34 CASSLASYTEA

NP-89-213 CASLLRGVRRAVL

NP-94-34 CASSSGQAYNS

NP-95-184 CASSSANYGY

CASSLTGTNEK

LCASKEGAGELF

CASSLSHRPATSST

CASSEWGAAYNEQ

CASSRGGRHNEQF

CASSLPDRGFSRETQY

CASSLYGPYNE

LCSVEETDKQYF

CASSLDRDNTGE

CASSLASYTEA

CASSSTSLNEKL

CASSLELAKN

CASSLEGSYEQY

CASSLELAKN

CASSLEGSYEQY

McFarland, EJ, Harding PA, Striebich CC, McWhinney S, Kuritzkes DR, Kotzin BL. Clonal CD8+ T Cell expansions in peripheral blood from human immunodeficiency virus type 1-Infected children. JID 2002: 186, 477-485. (3 patients; 23 sequences)

P011 CASSLFAGVSYNEQVFFG

P011 CASSPYSKQPZHFG

P011 CASGRLAELYNEQFFG

P011 CASSPSLAGETQUYFG

P011 CASSLYGLAGVKTYEQYFG

P099 CASSYLPGRYNSPLHFG

P099 CASSVVTGSGNYGYTFG

P099 CASSPATGTAHGYTFG

P099 CASSPITGTGSYGYTFG

P099 CASSYTPRTFYTEAFFG

P099 CASRLRETLSPLHFG

P099 CASSYARTGGTEAFFG

P099 CASSYSFLDRAYEQYFG

P099 CASSPRQPPYEQYFG

P111 CASSPMGVNEQFFG   (HIV-1 Env and Gag protein stimulated)

P111 CATTSWTSGALTGELFFG

P111 CASGRLAESSGANVLTEG

P111 CASSTASGGATDTQFG   (Gag protein stimulated)

P111 CASSTFDETDQPQHFG

P111 CASSALAGAYTDTQYFG

P111 CASIGDYGYNEQFFG

P111 CASSIVEGANEQFFG

P111 CASGRLAESSGANVLTFG   (Gag protein stimulated)

Weekes MP, Carmichael AJ, Wills MR, Mynard K, Sissons JGP. Human CD38-CD8+ T cells contain greatly expanded functional virus-specific memory CTL clones. J Immunol 1999; 162: 7569-7577. (3 patients; 11 sequences)

H0009	CASSYTLDNQPQH	(Env protein stimulated)
H0009	CASSLGSYEQY	(Gag protein stimulated)
H0018	CASTSGWGSYEQY	(Gag protein stimulated)
H0018	CASNLAPYEQY	(Gag protein stimulated)
H0018	CASSHDRQGASSPLH	(CMV pp65 stimulated)
H0018	CASSERTGGSYEQY	(CMV pp65 stimulated)
H0045	CASRQGAPNQPQH	(Env protein stimulated)
H0045	CASSDPLNEAF	(Env protein stimulated)
H0045	CASSLGGLF	(Env protein stimulated)
H0045	CASGMTQY	(Env protein stimulated)
H0045	CASSPIQGYYEQY	(CMV pp65 stimulated)

Kou ZC, Puhr JS, Wu SS, Goodenow MM, Sleasman JW. Combination antiretroviral therapy results in rapid increase in T cell receptor variable region beta repertoire diversity within CD45RA CD8 T cells in human immunodeficiency virus-infected children. JID 2003; 187: 385-395. (1 patient; 45 Sequences)

CASSLTGGNEQFFGPG

CASSPRGTDTQYFGPG

CASSSGLDDNEQFFGPG

CASSPTDSTDTQYFGPG

CASSSDTRGIQPQHFGDG

CASSPPRQGSYNEQFFGPG

CASSGEDRGTGQPGQFGDG

CASSPRGFGQGGETQYFGPG

CASSLTGGNEQFFGPG

CASSPSGSSYEQYFGPG

CASSSEDRRRIQPQHFGDG

CASSPGSPAEANYGYTFGSG

CAGQGGVSYEQYFGPG

CASSEASGVSEQYFGPG

CASSEFRTGGTNIQYFGAG

CASRLNEKYTFGSG

CASTEHFYYEQYFGPG

CASLREHNTEAFFGQG

CASSEGTGGLEQYFGPG

CASSEEAGTYEAFFGPG

CASRTFTGLQETQYFGPG

CASTTETAYNSPLHGFNG

CASRDYGGTGETQYFGPG

CASRARRGGHSYEQYFGPG

CASSGAGQGAVSEAFFGPG

CASSTPGTEHGTEAFFGPG

CASSVDRVKAFFGQG

CASSPDRGLAFFGPG

CASSQRRIGELFFGEG

CASSGRLARNEQFFGPG

CASSQDRSSTDTQYFGPG

CASSVDRVKAFFGQG

CASSQRTRGEQFRPG

CASSQDYHGEQYFGPG

CASSPGGLIEAFFGQG

CASSQEGLLGNEQFFGPG

CASSQEWRSSYEQYFGPG

CASSLGEGNTGELFFGPG

CASSQENSGGLYEEQYFGPG

CASSQERASDSSSYNEQFFGPG

CASSQDYHGEQYFGPG

CASSQDPGGTEAFFGQG

CASSATTGYNSPLHFGNG

CASSQERGTNYGYTFGSG

CASSQERASDSSSYNEQFFGPG

RANDOM TCR SEQUENCE LIST

>random sequence 1 consisting of 15 residues.

GSPEMQECCQAPDLA

>random sequence 2 consisting of 15 residues.

VMHGGNLVRQLYPQT

>random sequence 3 consisting of 15 residues.

RYSHTRARYEFSNRA

>random sequence 4 consisting of 15 residues.

GTDGACFHWFPVAQH

>random sequence 5 consisting of 15 residues.

CHSLGNQTLDEEDCM

>random sequence 6 consisting of 15 residues.

TTRAWMNMHMCCCAF

>random sequence 7 consisting of 15 residues.

NAVNTRKIRHQNQFE

>random sequence 8 consisting of 15 residues.

RIPLRLCCSTPGIFQ

>random sequence 9 consisting of 15 residues.

EEPSFWFCCQRANTR

>random sequence 10 consisting of 15 residues.

VRIVWGVMKYYNQPY

>random sequence 11 consisting of 15 residues.

ATCRFSHNHRMFQAK

>random sequence 12 consisting of 15 residues.

DRFWGAEIPAPADSN

>random sequence 13 consisting of 15 residues.

SKRAEVVHELICMIV

>random sequence 14 consisting of 15 residues.

RPCWEAMRKCNFVFH

>random sequence 15 consisting of 15 residues.

PNWAAVNCISCRPDS

>random sequence 16 consisting of 15 residues.

LESGCNACPALKAQD

>random sequence 17 consisting of 15 residues.

CCRLTSMIKQMVSKN

>random sequence 18 consisting of 15 residues.

IDMDHDEVSQVQVDK

>random sequence 19 consisting of 15 residues.

DYSAPHNEINGEVLW

>random sequence 20 consisting of 15 residues.

MSQWDHGSMQADHLK

>random sequence 21 consisting of 15 residues.

RVFWLPSYHEQQQPL

>random sequence 22 consisting of 15 residues.

RCDCYCIAFYKRNEN

>random sequence 23 consisting of 15 residues.

YPYNCCDQFLGKGFW

>random sequence 24 consisting of 15 residues.

KCECLCSKNSCPMEK

>random sequence 25 consisting of 15 residues.

GRCQNFMHFWKIDKP

>random sequence 26 consisting of 15 residues.

QWDDQCPRMHYIKNT

>random sequence 27 consisting of 15 residues.

IPNLNTERYRCCCPL

>random sequence 28 consisting of 15 residues.

YDQEVRTVTWDGVAF

>random sequence 29 consisting of 15 residues.

RFNDHEQSYCPMHYT

>random sequence 30 consisting of 15 residues.

EACAWRQNFSGFLWM

>random sequence 31 consisting of 15 residues.

QHPCSQVPVHKSSNF

>random sequence 32 consisting of 15 residues.

VKYDFTQKGTHDFEF

>random sequence 33 consisting of 15 residues.

QYNMLRGAEAMQFSL

>random sequence 34 consisting of 15 residues.

IIMIVHLSLARFWWQ

>random sequence 35 consisting of 15 residues.

AGQCWGCSQMAWNFT

>random sequence 36 consisting of 15 residues.

PVYVKYPDNFHVWNG

>random sequence 37 consisting of 15 residues.

SVANLLERSSSNVLV

>random sequence 38 consisting of 15 residues.

PARMNTCVCTKWVKM

>random sequence 39 consisting of 15 residues.

WVLKYRTGGFHWKTV

>random sequence 40 consisting of 15 residues.

TQNMQQPVRHKYWVE

>random sequence 41 consisting of 15 residues.

DRQHCMINTYVEHRD

>random sequence 42 consisting of 15 residues.

VFGNVVVQCAGWYSS

>random sequence 43 consisting of 15 residues.

RESLDGWGHRCRRGP

>random sequence 44 consisting of 15 residues.

EWMIFTNTDKRWALA

>random sequence 45 consisting of 15 residues.
QQWPCLTDMIHGHTY

>random sequence 46 consisting of 15 residues.
ATENWEPQSFAIANF

>random sequence 47 consisting of 15 residues.
AKLAHVSRSGVMALL

>random sequence 48 consisting of 15 residues.
CWIECCAPSPASFHC

>random sequence 49 consisting of 15 residues.
CYTVCFEQMEDKWRR

>random sequence 50 consisting of 15 residues.
TDRDYHLHVFPRTPY

>random sequence 51 consisting of 15 residues.
LDVVWSFTGRFSSTR

>random sequence 52 consisting of 15 residues.
AHDVENKYHTMRVRY

>random sequence 53 consisting of 15 residues.
ACAQKTIYREFEVRS

>random sequence 54 consisting of 15 residues.
HVVTGQVNFDHWWAY

>random sequence 55 consisting of 15 residues.
YAGFWDVGLWDLNWC

>random sequence 56 consisting of 15 residues.
HRVVEGPANWTVAIT

>random sequence 57 consisting of 15 residues.
CKMDTQHDHQGGYRW

>random sequence 58 consisting of 15 residues.
RPVDRASFCPQDWDD

>random sequence 59 consisting of 15 residues.
VRKYYEITPGMWMDH

>random sequence 60 consisting of 15 residues.
NEGYQCLNKEWGKRH

>random sequence 61 consisting of 15 residues.
PNYHNCKQNECQHNT

>random sequence 62 consisting of 15 residues.
YAQMAYFGCNTSTRQ

>random sequence 63 consisting of 15 residues.
DGRTDKEHADGSLAW

>random sequence 64 consisting of 15 residues.
AREMCIHDSQSKKEM

>random sequence 65 consisting of 15 residues.
YWRRTDAHEVLWIYT

>random sequence 66 consisting of 15 residues.
YEPWKTAVWMPAPNY

>random sequence 67 consisting of 15 residues.
VNNTWIIECDDPLFY

>random sequence 68 consisting of 15 residues.
RCHQCFMHSFYNWLT

>random sequence 69 consisting of 15 residues.
HMYLFQKSWWKCDWR

>random sequence 70 consisting of 15 residues.
KHSHRKATLVVLYLW

>random sequence 71 consisting of 15 residues.
HGPGSTAPFTPQNNL

>random sequence 72 consisting of 15 residues.
KPCDLWENCLKYMRA

>random sequence 73 consisting of 15 residues.
KHEYRPITSMNGHKH

>random sequence 74 consisting of 15 residues.

TTPLCINRYGQICVH

>random sequence 75 consisting of 15 residues.

RKQHSAKRMRWNNLW

>random sequence 76 consisting of 15 residues.

MLAVRDDWCMTKFDM

>random sequence 77 consisting of 15 residues.

EILNIVGYQHGCGKT

>random sequence 78 consisting of 15 residues.

VEYTVGCIERSVRLW

>random sequence 79 consisting of 15 residues.

WCQGHNLIIGMCKWS

>random sequence 80 consisting of 15 residues.

CWCNTLHHRRHMNLC

>random sequence 81 consisting of 15 residues.

AAPNAMFAYCYLVRI

>random sequence 82 consisting of 15 residues.

NEVGGRLQDEDGTGD

>random sequence 83 consisting of 15 residues.

SAVMVGSIQAFSACP

>random sequence 84 consisting of 15 residues.

CRIFTDNGDCQEFMF

>random sequence 85 consisting of 15 residues.

HKEQSHRPMNKHAYQ

>random sequence 86 consisting of 15 residues.

FTCACAKQADRHTGV

>random sequence 87 consisting of 15 residues.

MFHAQNKYPQKPFNE

>random sequence 88 consisting of 15 residues.

TPWYNDISNYAMSTV

>random sequence 89 consisting of 15 residues.
HHITSVFTTLEIYEV

>random sequence 90 consisting of 15 residues.
MAQAKVPFNERIPAD

>random sequence 91 consisting of 15 residues.
YMGAGGAKCEQCSYH

>random sequence 92 consisting of 15 residues.
EKCKHRMQALGPKRA

>random sequence 93 consisting of 15 residues.
VCWVYTIMGLQNTSS

>random sequence 94 consisting of 15 residues.
VMGQYGRQKYETFWS

>random sequence 95 consisting of 15 residues.
VCINFLIIKGFLPTA

>random sequence 96 consisting of 15 residues.
GVHLWTWHFPVAGVQ

>random sequence 97 consisting of 15 residues.
NEGMYDDIVLFMQNW

>random sequence 98 consisting of 15 residues.
PKITPHTDCPFPQQV

>random sequence 99 consisting of 15 residues.
RGWHVWGAPNLYRRQ

>random sequence 100 consisting of 15 residues.
ETPAPKHRNQQCVRP
